# Interference screws manufactured from magnesium display similar primary stability for soft tissue anterior cruciate ligament graft fixation compared to a biocomposite material – a biomechanical study

**DOI:** 10.1186/s40634-023-00663-3

**Published:** 2023-10-10

**Authors:** Adrian Deichsel, Johannes Glasbrenner, Michael J. Raschke, Matthias Klimek, Christian Peez, Thorben Briese, Elmar Herbst, Christoph Kittl

**Affiliations:** Department of Trauma, Hand and Reconstructive Surgery, University Hospital Münster, Albert-Schweitzer-Campus, Building W1, 48149 Münster, Germany

**Keywords:** Interference screw, Biocomposite, Magnesium, Acl reconstruction, Biomechanics

## Abstract

**Purpose:**

Biodegradable interference screws (IFS) can be manufactured from different biomaterials. Magnesium was previously shown to possess osteoinductive properties, making it a promising material to promote graft-bone healing in anterior cruciate ligament reconstruction (ACLR). The purpose of this study was to compare IFS made from magnesium to a contemporary biocomposite IFS.

**Methods:**

In a porcine model of ACL reconstruction, deep porcine flexor tendons were trimmed to a diameter of 8 mm, sutured in Krackow technique, and fixed with either 8 × 30 mm biocomposite IFS (Bc-IFS) or 8 × 30 mm magnesium IFS (Mg-IFS) in an 8 mm diameter bone tunnel in porcine tibiae. Cyclic loading for 1000 cycles from 0 to 250 N was applied, followed by load to failure testing. Elongation, load to failure and stiffness of the tested constructs was determined.

**Results:**

After 1000 cycles at 250 N, elongation was 4.8 mm ± 1.5 in the Bc-IFS group, and 4.9 mm ± 1.5 in the Mg-IFS group. Load to failure was 649.5 N ± 174.3 in the Bc-IFS group, and 683.8 N ± 116.5 in the Mg-IFS group. Stiffness was 125.3 N/mm ± 21.9 in the Bc-IFS group, and 122.5 N/mm ± 20.3 in the Mg-IFS group. No significant differences regarding elongation, load to failure and stiffness between Bc-IFS and Mg-IFS were observed.

**Conclusion:**

Magnesium IFS show comparable biomechanical primary stability in comparison to biocomposite IFS and may therefore be an alternative to contemporary biodegradable IFS.

## Introduction

Interference screws (IFS) are the most frequently used implants for tunnel aperture fixation of soft tissue anterior cruciate ligament (ACL) grafts [7]. While originally made from nondegradable materials like titanium alloys, contemporary IFS are mostly made from biodegradable materials. These suggest reliable graft fixation, as well as subsequent resorption, omitting the need for implant removal, especially in case of revision [29]. Different biomaterials like biopolymers, or biocomposites are available, each with different biological and mechanical properties [33]. However, for biodegradable IFS, incomplete resorption, or replacement with insufficient bone material are described [5, 32], which may cause inflammatory reactions with possible detrimental effect for the patient.

A possible alternative material for biodegradable IFS may be magnesium (Mg). It closely resembles the stiffness of human bone and was shown to elicit osteoinductive effects, both in vitro and in vivo, which may facilitate a quicker graft healing in ACL reconstruction [8, 40, 42, 47, 49]. Implants made of pure Mg have already been used in trauma surgery, but suffered from certain drawbacks. One of which was the unpredictable speed of degradation, which caused implant failure before adequate healing could be obtained [2, 47]. Furthermore, release of hydrogen gas during degradation may lead to osteolysis and formation of gas caverns [25, 30]. Due to these disadvantages, different solutions, including alloying magnesium with other metals, surface modifications such as coating or ceramization, were introduced to control the degradation process and to better suit these implants for orthopedic and trauma surgery [2, 20, 21, 39]. Even though magnesium IFS (Mg-IFS) are already purchasable, there is limited data on their biomechanical properties [15].

Thus, the purpose of this study was to investigate the biomechanical primary stability of ACL graft fixation with a novel Mg-IFS, and to compare it to a biocomposite IFS (Bc-IFS). We hypothesized that Mg-IFS show equivalent primary stability for ACL graft fixation.

## Materials and methods

### Ethics statement

Frozen (-20 °C) Porcine knees and lower legs were obtained from a local butcher, who confirmed adequate health and comparable age of all used animals. Magnesium IFS were kindly provided by Medical Magnesium GmbH (Aachen, Germany). All other implants and materials were commercially purchased. Ethical approval was waived by the institutional review board of our institute.

### Graft fixation strategies

**Fig. 1:**
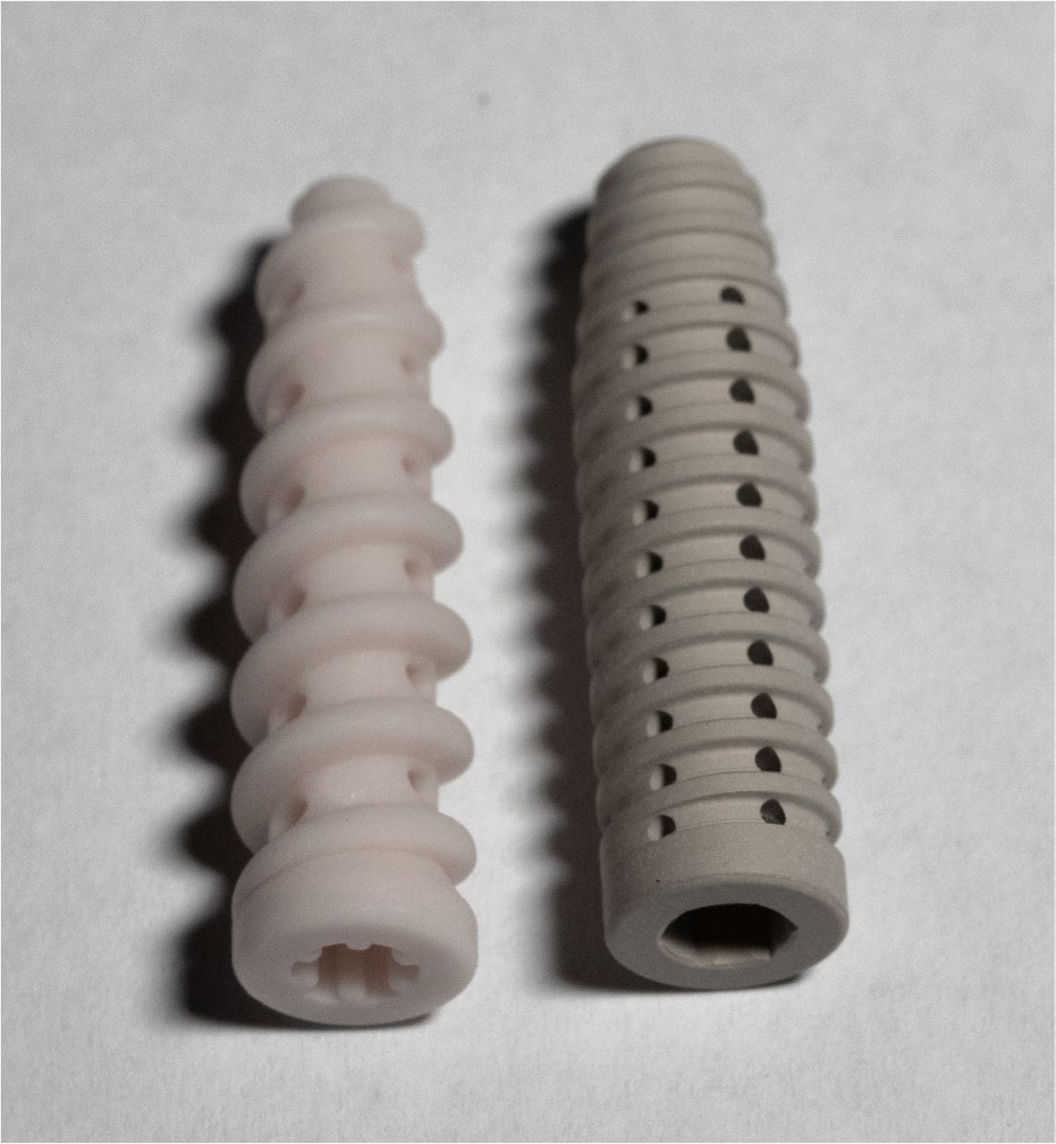
Implants utilized for graft fixation. Left: 8 × 30 mm FastThread™ biocomposite interference screw (Arthrex, Inc); right: 8 × 30 mm magnesium interference screw (Medical Magnesium GmbH)

**Fig. 2:**
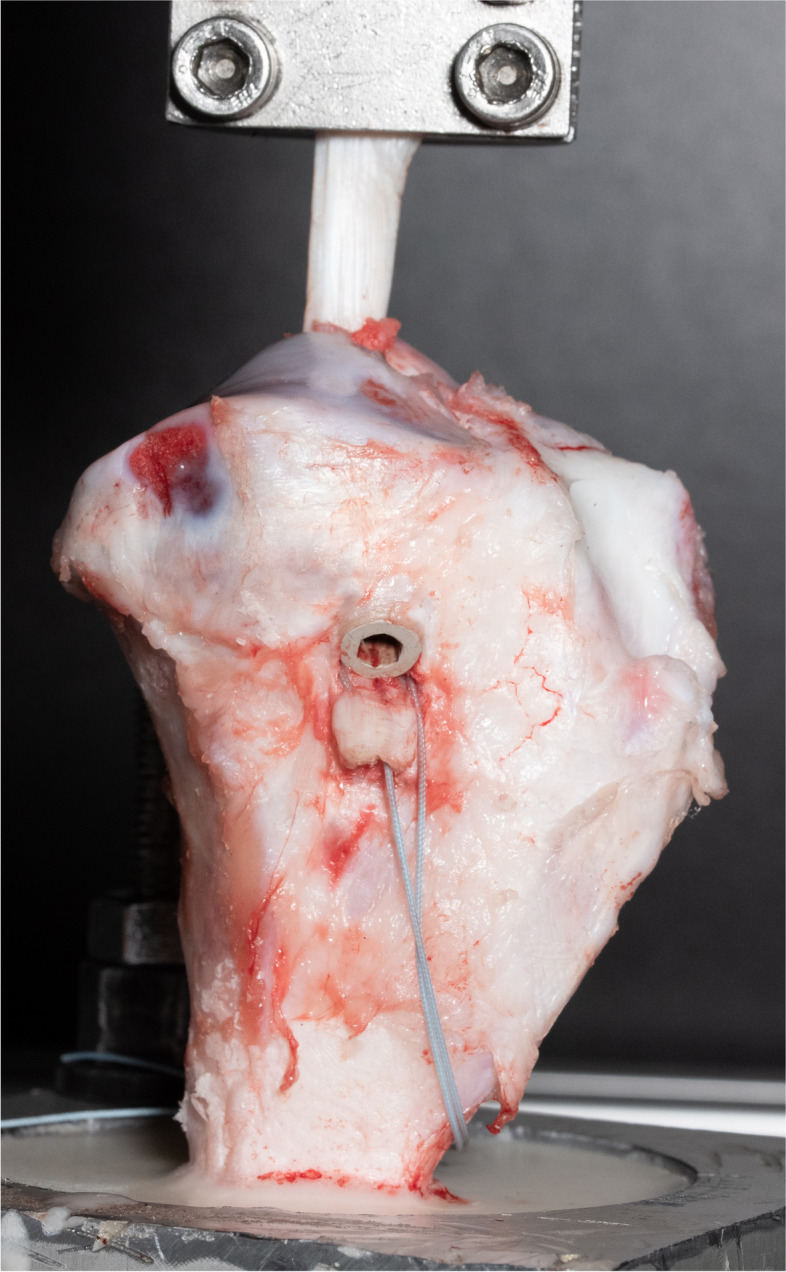
Graft fixation. Deep porcine flexor tendons with a diameter of 8 mm were sutured in Krackow technique and fixed with an 8 mm interference screw in an 8 mm bone tunnel in porcine tibial bone

The following implants were used and compared in this study (Fig. 1): For interference screw fixation, 8 × 30 mm *FastThread*™ biocomposite (30% biphasic calcium phosphate, and 70% poly L-lactide-co-D acid) IFS (Bc-IFS; Arthrex, Inc) and 8 × 30 mm mm*.IF* WE43MEO (alloy containing Yttrium, Zirconium, and rare earths) magnesium IFS (Mg-IFS; Medical Magnesium GmbH) were used (each *n* = 10). Porcine knees were defrosted at 7 degrees Celsius for 24 h (no repeated freezing and thawing), muscles and soft tissue were removed, and the tibia embedded into an aluminum mount using synthetic resin (RenCast® FC 52/53 A ISO and Ren Cast® FC 53 B Polyol, Gößl & Pfaff). In case of visible osteoarthritis, or fracture of the specimen, it was excluded. Deep porcine flexor tendons were prepared from porcine hind feet and trimmed to a diameter of 8 mm and a length of 100 mm, to resemble the thickness of a quadriceps ACL graft typically used in the clinical setting [38]. The diameter of the tendons was measured using a standardized sizing device (± 0.5 mm, Karl Storz). The distal 20 mm of the tendon grafts were sutured in Krackow technique with four stiches on each side using high strength polyethylene suture (FiberWire® #2, Arthrex, Inc). An 8 mm bone tunnel with a length of 5 cm and an angulation of approximately 50° was drilled through the anatomical insertion site of the native ACL. The graft was then shuttled through the tunnel and fixed with one of the two IFS (Fig. 2). Envelope randomization was used to determine the implant used for each test.

### Biomechanical testing

For biomechanical testing, a servo-hydraulic uniaxial testing machine (Model 8874, Instron), equipped with a 0 to 20 kN sensor, was used. The mount containing the embedded porcine tibia was fixed to the base of the machine with two clamps. The proximal end of the graft was fixed 20 mm above the tibial joint line to the testing machine using a cryoclamp, simulating the intra-articular part of the graft. Before starting the experiment, the graft was manually pretensioned to 20 N [19]. All constructs were first exposed to cyclic loading, followed by a load to failure test. For testing of the tibial graft-IFS-construct, the following protocol was used: Preconditioning was performed using 10 cycles at 50 N and cyclic loading was performed at a frequency of 0.5 Hz. Then, 1000 cycles with a load from 30 to 250 N per cycle were performed, as previously described [17]. Subsequently, load to failure testing was performed at a speed of 25 mm/min. Elongation and load were recorded continuously during the entire test. Stiffness was calculated by the slope of the linear portion of the load–displacement curve during load to failure testing. The mode of failure was macroscopically documented, and the tested IFS was visually inspected for damage to the implant.

### Data analysis

An a priori power analysis was performed using G*Power 3.1 (university of Düsseldorf, Germany) to calculate the sample size needed for this study [16]. To detect a difference of 100 N between group means at a standard deviation of 60 N, a sample size of n = 10 per group was calculated, to obtain a power of at least 90%. The assumed standard deviation (SD) was based on previously reported studies on graft fixation strategies in porcine knee models [15]. Extraction of biomechanical parameters from test data was performed using Matlab (R2020a, MathWorks). Statistical analysis was performed using PRISM (version 8, GraphPad Software). The results are presented as mean values and corresponding standard deviations (SD). The Distribution of the data for each variable was assessed utilizing histograms, as well as the Shapiro–Wilk test. Since not all groups fulfilled the criteria for normal distribution, statistical comparison was performed using the Mann–Whitney test. A *p*-value ≤ 0.05 was defined as a significant difference.

## Results

Both IFS were able to withstand the torques during insertion without breakage. None of the IFS were damaged during insertion into the bone tunnel.

After 1000 cycles at 250 N, elongation was 4.8 ± 1.5 mm in the Bc-IFS group, and 4.9 ± 1.5 mm in the Mg-IFS group (Table 1). No significant differences between Bc-IFS and Mg-IFS were observed. Load to failure was 649.5 ± 174.3 N in the Bc-IFS group, and 683.8 ± 116.5 N in the Mg-IFS group. Stiffness was 125.3 ± 21.9 N/mm in the Bc-IFS group, and 122.5 ± 20.3 N/mm in the Mg-IFS group. No significant differences regarding elongation, load to failure and stiffness between Bc-IFS and Mg-IFS were observed.

**Table 1:** Summary of elongation, load to failure, stiffness

	Elongation [mm]		Load to failure [N]		Stiffness [N/mm]	
	Mean	SD	Mean	Mean	Mean	SD
Bc-IFS	4.8	± 1.5	649.5	± 174.3	125.5	± 21.9
Mg-IFS	4.9	± 1.5	683.8	± 116.5	122.5	± 20.3

*Bc-IFS* Biocomposite interference screw, *Mg-IFS* Magnesium interference screw

**Fig. 3:**
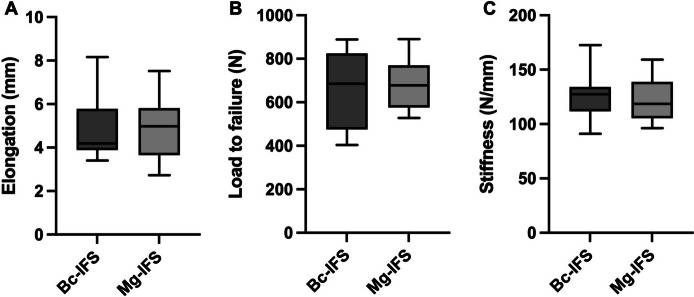
Biomechanical primary (time-zero) stability. Elongation after 1000 cycles at 250 N (**A**), load to failure (**B**), and stiffness (**C**), as boxplots presenting mean, standard deviation and spread; Bc-IFS = biocomposite interference screw, Mg-IFS = magnesium interference screw

Mode of failure was tendon pullout in 8 specimens of the Bc-IFS group, and in 7 of the Mg-IFS group. In the rest of the cases, the constructs failed by elongation of the graft, followed by a rupture at the proximal tunnel aperture. However, elongation and load to failure between the different failure modes did not differ. There was no visual slippage at the cryoclamp and no damage to the cortex, or fracture of the bone in any specimen, after load to failure testing (Fig. 3).

## Discussion

The most important finding of this study was that IFS made from magnesium have similar biomechanical primary stability, compared to a contemporary IFS made from a biocomposite material. The loads acting on the ACL during a gait cycle and other everyday activities like sitting and squatting were reported to range from 0 to 454 N [26, 27, 37]. During early rehabilitation with limited weight bearing, these loads are likely to be further reduced. Both tested IFS were shown to withstand these reported loads.

Primary stability of magnesium IFS was previously compared to other materials in few biomechanical studies. In a robotic biomechanical study, utilizing cadaveric knee specimen, IFS made from pure magnesium were compared to polylactic acid IFS for ACL graft fixation, in a human cadaveric knee model of ACL reconstruction [36]. No significant differences in anterior tibial translation in different flexion angles, when ACL reconstructions were fixed with either of the screws, were found. In another biomechanical study, utilizing a porcine model of femoral graft fixation, 8 × 23 mm IFS made from a MgYREZr (alloy containing yttrium) alloy showed equivalent primary stability in comparison to a 8 × 23 PEEK screw, with load to failure values of 529.0 ± 63.3 N vs. 511.3 ± 66.5 N, and elongation of 5.1 ± 0.5 mm vs. 5.1 ± 0.4 mm, comparable to the results of the present study [15]. Further studies compared different biodegradable IFS to titanium IFS, with no significant differences between biodegradable and titanium implants, regarding both biomechanical primary stability, as well as clinical outcome [9, 14, 29, 46]. Exemplary, Weiler et al. compared six different biodegradable IFS to a titanium IFS, finding mean load to failure values ranging from 439.2 to 830.2 N for the biodegradable implants, compared to 821.6 ± 129.8 N for the titanium implant [46]. These results, although not directly comparable due to differences in biomechanical testing and experimental setup, are similar to the findings of the present study, which reported load to failure of 683.9 ± 116.5 N for the tested Mg-IFS. This suggests that the tested WE43 Mg-IFS possesses adequate primary stability for ACL reconstruction, comparable to that of other biodegradable IFS.

The integration of a tendon graft into the bone tunnel is known to undergo different phases during which the graft remodels and attaches to the tendon-bone interface [35, 36, 45]. The choice of implant for fixation is known to influence the tendon-bone integration and biomechanical properties during the course of healing [13, 44, 45, 48]. Magnesium IFS were previously shown to improve tendon-bone healing in multiple small animal studies, with favorable results in comparison to both titanium IFS as well as extracortical tendon fixation [8, 11, 42, 43]. Despite these possible advantages, previous generations of magnesium implants suffered from unpredictable speed of degradation [23], as well as intraosseous osteolysis and formation of gas caverns, caused by nitrogen release during corrosion of the implant [25, 30, 41]. Even though degradation speed of newer generations of Mg -IFS has been controlled by alloying magnesium with other metals [22–24], and surface treatment like ceramization, or plasma electrolytic oxidation (PEO) [21, 34], surgeons should still be aware of these possible disadvantages. The Mg-IFS used in this study were made from a WE43MEO alloy, containing Yttrium, Zirconium, and rare earths. WE43 alloys were previously shown to start degradation between the 12th and 16th week in a small animal model, and exhibited favorable degradation kinetics, in comparison to a PLLA implant [19, 20]. PEO coating, as used with the tested Mg-IFS, was shown to decrease the degradation rate and hydrogen gas release in fluid immersion experiments [3, 4]. However, relevant large animal studies and clinical trials are required, to determine in-vivo degradation kinetics, before routinely using the newer generation Mg-IFS for aperture fixation in ACL reconstruction.

This biomechanical study has several limitations to mention. Previous studies showed that the porcine knee anatomy and ligament biomechanics are adequately similar to humans, making it a frequently used model to assess biomechanical primary stability of orthopedic implants [10, 12, 17, 31]. However, bone density in the porcine model is significantly higher in comparison to humans [1]. Since load to failure of IFS fixation was shown to be dependent on the bone mineral density, this could have biased the load to failure towards higher values [6, 28]. Furthermore, freezing and thawing of the porcine knees might have influenced the mechanical properties of the specimens [18]. A Bc-IFS was used as control group in the present study, to compare Mg-IFS to another biodegradable implant. Biodegradable IFS were shown to provide comparable fixation strength, in comparison to titanium IFS [14, 46]. The IFS utilized in this study have different geometries, most prominently different thread shapes and thread pitches, which could have possibly influenced the biomechanical properties of the screws. However, both geometries reflect implants currently available in clinical practice, and thread shape was shown not to influence biomechanical properties of interference screws [17]. The comparisons between the implants are limited to the timepoint zero. Possible influences of the different materials on tendon-bone healing cannot be deduced. Therefore, clinical trials or large animal models are needed to investigate the influence of the material on the course of healing after ACL reconstruction.

## Conclusion

Magnesium IFS show comparable biomechanical primary stability in comparison to biocomposite IFS and may therefore be an alternative to contemporary biodegradable IFS.

## Data Availability

Data will be made available on reasonable request.

## References

[1] Aerssens J, Boonen S, Lowet G, Dequeker J (1998). Interspecies differences in bone composition, density, and quality: potential implications for in vivo bone research*. Endocrinology.

[2] Amukarimi S, Mozafari M (2021). Biodegradable magnesium-based biomaterials: An overview of challenges and opportunities. MedComm.

[3] Arrabal R, Matykina E, Viejo F, Skeldon P, Thompson GE (2008). Corrosion resistance of WE43 and AZ91D magnesium alloys with phosphate PEO coatings. Corros Sci.

[4] Barati Darband G, Aliofkhazraei M, Hamghalam P, Valizade N (2017). Plasma electrolytic oxidation of magnesium and its alloys: Mechanism, properties and applications. J Magnesium Alloys.

[5] Barber FA, Dockery WD (2020). Biocomposite interference screws in anterior cruciate ligament reconstruction: osteoconductivity and degradation. Arthrosc Sports Med Rehabil.

[6] Brand JC, Pienkowski D, Steenlage E, Hamilton D, Johnson DL, Caborn DN (2000). Interference screw fixation strength of a quadrupled hamstring tendon graft is directly related to bone mineral density and insertion torque. Am J Sports Med.

[7] Budny J, Fox J, Rauh M, Fineberg M (2017). Emerging Trends in Anterior Cruciate Ligament Reconstruction. J Knee Surg.

[8] Cheng P, Han P, Zhao C, Zhang S, Zhang X, Chai Y (2016). Magnesium inference screw supports early graft incorporation with inhibition of graft degradation in anterior cruciate ligament reconstruction. Sci Rep.

[9] Debieux P, Franciozi CE, Lenza M, Tamaoki MJ, Magnussen RA, Faloppa F (2016). Bioabsorbable versus metallic interference screws for graft fixation in anterior cruciate ligament reconstruction. Cochr Database Syst Rev.

[10] Deichsel A, Raschke MJ, Herbst E, Peez C, Oeckenpöhler S, Briese T (2022). The biomechanical stability of bone staples in cortical fixation of tendon grafts for medial collateral ligament reconstruction depends on the implant design. Am J Sports Med.

[11] Diekmann J, Bauer S, Weizbauer A, Willbold E, Windhagen H, Helmecke P (2016). Examination of a biodegradable magnesium screw for the reconstruction of the anterior cruciate ligament: A pilot in vivo study in rabbits. Mater Sci Eng C.

[12] Domnick C, Wieskötter B, Raschke MJ, Schulze M, Kronenberg D, Wefelmeier M (2016). Evaluation of biomechanical properties: are porcine flexor tendons and bovine extensor tendons eligible surrogates for human tendons in in vitro studies?. Arch Orthop Trauma Surg.

[13] Ekdahl M, Wang JH, Ronga M, Fu FH (2008). Graft healing in anterior cruciate ligament reconstruction. Knee Surg Sports Traumatol Arthrosc.

[14] Ettinger M, Schumacher D, Calliess T, Dratzidis A, Ezechieli M, Hurschler C (2014). The biomechanics of biodegradable versus titanium interference screw fixation for anterior cruciate ligament augmentation and reconstruction. Int Orthop.

[15] Ezechieli M, Meyer H, Lucas A, Helmecke P, Becher C, Calliess T (2016). Biomechanical Properties of a Novel Biodegradable Magnesium-Based Interference Screw. Orthop Rev (Pavia).

[16] Faul F, Erdfelder E, Buchner A, Lang A-G (2009). Statistical power analyses using G*Power 3.1: Tests for correlation and regression analyses. Behav Res Methods.

[17] Garcés GL, Martel O, Yánez A, Cuadrado A (2019). Does thread shape affect the fixation strength of the bioabsorbable interference screws for anterior cruciate ligament reconstructions? A biomechanical study. BMC Musculoskeletal Disord.

[18] Giannini S, Buda R, Di Caprio F, Agati P, Bigi A, De Pasquale V (2008). Effects of freezing on the biomechanical and structural properties of human posterior tibial tendons. Int Orthop.

[19] Glasbrenner J, Deichsel A, Raschke MJ, Briese T, Frank A, Herbort M (2021). Bone staples provide favorable primary stability in cortical fixation of tendon grafts for medial collateral ligament reconstruction: a biomechanical study. Orthop J Sports Med.

[20] Haghshenas M (2017). Mechanical characteristics of biodegradable magnesium matrix composites: a review. J Magnesium Alloys.

[21] Jung O, Porchetta D, Schroeder M-L, Klein M, Wegner N, Walther F (2019). In vivo simulation of magnesium degradability using a new fluid dynamic bench testing approach. Int J Mol Sci.

[22] Levorova J, Duskova J, Drahos M, Vrbova R, Vojtech D, Kubasek J (2018). In vivo study on biodegradable magnesium alloys: bone healing around WE43 screws. J Biomater Appl.

[23] Luo Y, Zhang C, Wang J, Liu F, Chau KW, Qin L (2021). Clinical translation and challenges of biodegradable magnesium-based interference screws in ACL reconstruction. Bioactive Mater.

[24] Marukawa E, Tamai M, Takahashi Y, Hatakeyama I, Sato M, Higuchi Y (2016). Comparison of magnesium alloys and poly-l-lactide screws as degradable implants in a canine fracture model. J Biomed Mater Res B Appl Biomater.

[25] Meier R, Panzica M (2017). First results with a resorbable MgYREZr compression screw in unstable scaphoid fractures show extensive bone cysts. Handchir Mikrochir Plast Chir.

[26] Morrison JB (1969). Function of the knee joint in various activities. Biomed Eng.

[27] Noyes FR, Butler DL, Grood ES, Zernicke RF, Hefzy MS (1984). Biomechanical analysis of human ligament grafts used in knee-ligament repairs and reconstructions. J Bone Joint Surg Am.

[28] Nurmi JT, Sievänen H, Kannus P, Järvinen M, Järvinen TL (2004). Porcine tibia is a poor substitute for human cadaver tibia for evaluating interference screw fixation. Am J Sports Med.

[29] Papalia R, Vasta S, D'Adamio S, Giacalone A, Maffulli N, Denaro V (2014). Metallic or bioabsorbable interference screw for graft fixation in anterior cruciate ligament (ACL) reconstruction?. Br Med Bull.

[30] Plaass C, Ettinger S, Sonnow L, Koenneker S, Noll Y, Weizbauer A (2016). Early results using a biodegradable magnesium screw for modified chevron osteotomies. J Orthop Res.

[31] Proffen BL, McElfresh M, Fleming BC, Murray MM (2012). A comparative anatomical study of the human knee and six animal species. Knee.

[32] Radford MJ, Noakes J, Read J, Wood DG (2005). The natural history of a bioabsorbable interference screw used for anterior cruciate ligament reconstruction with a 4-strand hamstring technique. Arthroscopy.

[33] Ramos DM, Dhandapani R, Subramanian A, Sethuraman S, Kumbar SG (2020). Clinical complications of biodegradable screws for ligament injuries. Mater Sci Eng C Mater Biol Appl.

[34] Rendenbach C, Fischer H, Kopp A, Schmidt-Bleek K, Kreiker H, Stumpp S (2021). Improved in vivo osseointegration and degradation behavior of PEO surface-modified WE43 magnesium plates and screws after 6 and 12 months. Mater Sci Eng C Mater Biol Appl.

[35] Rodeo SA, Arnoczky SP, Torzilli PA, Hidaka C, Warren RF (1993). Tendon-healing in a bone tunnel. A biomechanical and histological study in the dog. J Bone Joint Surg Am.

[36] Scheffler SU, Unterhauser FN, Weiler A (2008). Graft remodeling and ligamentization after cruciate ligament reconstruction. Knee Surg Sports Traumatol Arthrosc.

[37] Shelburne KB, Torry MR, Pandy MG (2005). Muscle, ligament, and joint-contact forces at the knee during walking. Med Sci Sports Exerc.

[38] Snaebjörnsson T, Hamrin-Senorski E, Svantesson E, Karlsson L, Engebretsen L, Karlsson J (2019). Graft Diameter and Graft Type as Predictors of Anterior Cruciate Ligament Revision: A Cohort Study Including 18,425 Patients from the Swedish and Norwegian National Knee Ligament Registries. J Bone Joint Surg Am.

[39] Song G (2007). Control of biodegradation of biocompatable magnesium alloys. Corros Sci.

[40] Staiger MP, Pietak AM, Huadmai J, Dias G (2006). Magnesium and its alloys as orthopedic biomaterials: a review. Biomaterials.

[41] Thormann U, Alt V, Heimann L, Gasquere C, Heiss C, Szalay G (2015). The biocompatibility of degradable magnesium interference screws: an experimental study with sheep. Biomed Res Int.

[42] Wang J, Xu J, Fu W, Cheng W, Chan K, Yung PS-H (2017). Biodegradable magnesium screws accelerate fibrous tissue mineralization at the tendon-bone insertion in anterior cruciate ligament reconstruction model of rabbit. Sci Rep.

[43] Wang J, Xu J, Song B, Chow DH, Shu-Hang Yung P, Qin L (2017). Magnesium (Mg) based interference screws developed for promoting tendon graft incorporation in bone tunnel in rabbits. Acta Biomater.

[44] Weiler A, Hoffmann RF, Bail HJ, Rehm O, Südkamp NP (2002). Tendon healing in a bone tunnel. Part II: Histologic analysis after biodegradable interference fit fixation in a model of anterior cruciate ligament reconstruction in sheep. Arthroscopy.

[45] Weiler A, Peine R, Pashmineh-Azar A, Abel C, Südkamp NP, Hoffmann RF (2002). Tendon healing in a bone tunnel. Part I: Biomechanical results after biodegradable interference fit fixation in a model of anterior cruciate ligament reconstruction in sheep. Arthroscopy.

[46] Weiler A, Windhagen HJ, Raschke MJ, Laumeyer A, Hoffmann RF (1998). Biodegradable interference screw fixation exhibits pull-out force and stiffness similar to titanium screws. Am J Sports Med.

[47] Witte F, Hort N, Vogt C, Cohen S, Kainer KU, Willumeit R (2008). Degradable biomaterials based on magnesium corrosion. Curr Opin Solid State Mater Sci.

[48] Zantop T, Weimann A, Wolle K, Musahl V, Langer M, Petersen W (2007). Initial and 6 weeks postoperative structural properties of soft tissue anterior cruciate ligament reconstructions with cross-pin or interference screw fixation: an in vivo study in sheep. Arthroscopy.

[49] Zhang Y, Xu J, Ruan YC, Yu MK, O'Laughlin M, Wise H (2016). Implant-derived magnesium induces local neuronal production of CGRP to improve bone-fracture healing in rats. Nat Med.

